# The impact of human capital efficiency on Latin American mutual funds during Covid-19 outbreak

**DOI:** 10.1186/s41937-020-00066-6

**Published:** 2020-10-21

**Authors:** Nawazish Mirza, Jamila Abaidi Hasnaoui, Bushra Naqvi, Syed Kumail Abbas Rizvi

**Affiliations:** 1 Excelia Business School, La Rochelle, France; 2 Lahore University of Management Sciences, Lahore, Pakistan; 3 Lahore School of Economics, Lahore, Pakistan

**Keywords:** G01, G10, G11, G20, Covid-19, Equity funds, Latin America, Human capital

## Abstract

The mutual funds’ returns, inter alia, are dependent on fund managers’ performance. This makes human capital efficiency very central for consistent risk-adjusted performance. The persistence in performance becomes more critical during periods of high turbulence, like the one we are experiencing amidst the outbreak of Covid-19. In this research, we attempt to evaluate the performance of equity funds in massively impacted Latin American countries. These equity funds, with 95% of their investment in the infected region, are ranked as per their human capital efficiency using 2019 as the base year. Our findings demonstrate that funds with higher human capital efficiency significantly outperform their counterparts that rank lower on human capital efficiency. These findings remained consistent for the sub-periods that we specify to map the evolution of Covid-19. We conclude that equity funds should enhance their human capital efficiency to endure resilience amid macroeconomic shocks.

## Introduction

Human capital efficiency (HCE) is deemed critical for financial services. Nawaz (2019) reported a positive relationship between intellectual capital and market value of the banks. Meles, Porzio, Sampagnaro, and Verdoliva (2016) attributed the efficiency of commercial banks to their investment in intellectual capital in general and human capital in specific. Likewise, Joshi, Cahill, and Sidhu (2010) and Mention and Bontis (2013) also noted the relevance of human capital for the banking sector. Similar to the banking sector, the asset management industry is very much dependent on portfolio managers, and it is interesting to evaluate mutual funds’ investment in human capital translates into performance. Surprisingly, the evidence on the relationship between funds’ performance and human capital efficiency is scant. As suggested by Yarovaya, Mirza, Abaidi, and Hasnaoui (2020), in a rational setting, the funds with higher HCE should have superior performance to their counterparts. The issue is even more critical in the context of the Covid-19 outbreak when the global economic systems are in a rout, and portfolio managers are facing extreme performance pressures. Therefore, it is very relevant to observe if funds’ performance can be differentiated on the basis of their HCE.

There have been many recent studies that have documented the initial impact of Covid-19 on financial systems. These studies have reported an increase in systematic risk (Zhang, Hu, & Ji, 2020), a rise in market volatility due to policy interventions (Zaremba, Kizys, Aharon, & Demir, 2020), stock market contagion (Akhtaruzzaman, Boubaker, & Sensoy, 2020), and spillover across commodities and cryptocurrencies (Corbet, Larkin, & Lucey, 2020). For mutual funds, Mirza, Naqvi, Rahat, and Rizvi (2020) and Rizvi, Mirza, Naqvi, and Rahat (2020) found out that funds’ managers demonstrate volatility timing and drift in investment styles as an attempt to subside the impact of Covid-19. This evidences also reported positive performance for a selected category of mutual funds. The initial findings of the effects of Covid-19 have been concentrated mostly across China, the European Union, and the USA. This is plausible because the outbreak that initiated from China has its epicenter gradually shifted to European Union and the USA resulting in significant impairment of these economies. In the last few weeks, the world is now witnessing a new epicenter of Covid-19 with Latin America becoming the mass victim of this pandemic.

Although the first known case of Covid-19 in Latin America was reported on February 26 in Brazil, the situation started getting worse from May onwards. This culminated in an official pronouncement by the World Health Organization (WHO), declaring Latin America as the new epicenter since the region’s daily mortality rate surpassed Europe and the USA. The regional growth pre-Covid-19 was estimated to be below 2%. World Bank revised this in mid-April with an estimated contraction of 4.6%. As the pandemic deepens, the estimate was reviewed in June. In the most recent forecasts, the regional economies are expected to face a contraction of 7.2%^1^. While the local governments are poised to combat the spread through interventions, the Covid-19 episode is likely to take a toll on financial markets in the foreseeable future. These dynamics offer a unique opportunity to assess if the human capital efficiency translates into resilient and differentiated performance for Latin American mutual funds. The rest of the paper is organized as follows. Section 2 illustrates our data and methodology, results are presented in Section 3, and Section 4 provides some tentative conclusions.

## Data and methodology

We begin our analysis by selecting the sample of mutual funds from the Latin American countries that are most impacted by the outbreak of Covid-19. We consider countries that have reported more than 60,000 Covid-19 cases each as on July 7, 2020 (our cut-off date). This limits our sample to Brazil, Peru, Chile, Mexico, Colombia, Argentina, and Ecuador. The cumulative Covid-19 cases across these seven countries account for 24% of total global infections, while their reported deaths are around 23% of the world’s mortality from this pandemic. Six out of seven Latin American states have a death per million factors higher than the global average (68.9). Chile’s death per million statistics is maximum, with 330 deaths/million, and Argentina reporting the minimum of 33 deaths/million. Table 1 presents some statistics related to Covid-19 for selected countries.

**Table 1:** Covid-19 statistics for selected Latin American countries

Country	Total cases	Total deaths	Total recovered	Active cases	Tot cases/1 M pop.	Deaths/1 M pop.
World	11,564,185	536,893	6,538,868	4,488,424	1,484	68.9
Brazil	1,604,585	64900	978,615	561,07	7,548	305
Peru	302,718	10589	193,957	98,172	9,18	321
Chile	295,532	6308	261,032	28,192	15,458	330
Mexico	256,848	30639	155,604	70,605	1,992	238
Colombia	117,110	4064	47,881	65,165	2,301	80
Argentina	77,815	1507	27,597	48,711	1,722	33
Ecuador	61,958	4781	28,722	28,455	3,511	271

Source: https://www.worldometers.info/ (dated July 7, 2020)

The next step is to select mutual funds from these seven countries. To minimize the data accessibility issues (and availability of appropriate benchmarks), we limit our sample to equity funds. Following Pulic (2000) and Pulic and Kolakovic (2003), we express human capital efficiency (HCE) as

1 $$ \mathrm{HCE}=\frac{V_{\mathrm{A}}}{C_{\mathrm{H}}}, $$

where *V*_A_ is the value-added calculated as the product of the fund’s annualized alpha and asset under management. *C*_H_ corresponds to investment in human capital that incorporates various types of paid compensations and benefits. These include salaries, bonuses, commissions, incentives, traveling allowances, housing, medical, training, development, etc. While net asset values (NAVs) are mostly available, the details on funds compensation are not always publicly disseminated. Therefore, an essential criterion for the inclusion of funds in our sample is the availability of compensation-related data. Finally, to have a Latin American perspective and capture the local impact of Covid-19, we only include funds that have 95% of their investment within the region. Based on this, our final sample constitutes of 493 equity funds across the seven selected countries. We compute HCE for each fund for 2019 on December 31st (pre-Covid-19 period) using CAPM based alpha, assets under management, and investment in human capital. The computed HCE is used to rank these funds across five groups (20% each) from low to high HCE, and we assess their comparative performance across these sorts. We expect that the funds with higher HCE should perform better than the funds that have lower HCE. The country-wise sample distribution based on HCE rank is presented in Table 2.

**Table 2:** Sample distribution (HCE sort)

	Low	2	3	4	High	Total
Brazil	22	25	22	24	26	119
Peru	14	15	13	14	12	68
Chile	12	10	15	12	13	62
Mexico	13	12	11	12	11	59
Colombia	12	13	13	12	10	60
Argentina	15	12	15	14	12	68
Ecuador	11	11	12	11	12	57
Total	99	98	101	99	96	493

For this analysis, we consider a full period and multiple sub-periods. This is to establish the robustness of our results vis-à-vis the evolution of Covid-19. Our entire period spans from January 1 to July 7, 2020^2^. From January to March, the spread was modest within Latin America, while the outbreak was massive across Europe. Therefore, we define our first stage from January 1 to March 21. During this period, none of the sample countries reported more than 1000 individual cases. The second stage is from March 22 to May 3, during which none of the countries have reported more than a hundred thousand individual cases. The final stage was from May 4 to July 7 when the pandemic became furious, and as of July 7, there were more than 2.7 million cumulative cases in the region. This is 12.4× more than stage 2. These statistics are presented in Table 3.

**Table 3:** Phases of evolution of Covid-19 in Latin America

Stage	Cut-off dates	Cumulative cases	Description
1	January 1 to March 21, 2020	2546	The individual country cases did not exceed 1000
2	March 22 to May 3, 2020	219,142	The individual country cases did not exceed 100,000
3	May 4 to July 7, 2020	2,716,566	The massive outbreak across all countries

To measure comparative performance, we use different evaluation criteria. These include adjusted Sharpe ratio, Sortino ratio, Treynor ratio, and Information ratio. The adjusted Sharpe ratio was proposed by Pezier and White (2006). As documented by Mirza et al. (2020), Reddy, Mirza, Naqvi, and Fu (2017) and Rizvi et al. (2020), it accounts for possible non-normality in NAV based returns. The functional forms of fund’s returns (*R*_it_) and these measures are as follows:

2 $$ {R}_{\mathrm{it}}=\frac{{\mathrm{NAV}}_{\mathrm{it}}-{\mathrm{NAV}}_{\mathrm{it}-1}}{{\mathrm{NAV}}_{\mathrm{it}-1}}, $$

3 $$ \mathrm{Sharpe}\ \mathrm{Ratio}={\mathrm{SR}}_{\mathrm{i}}=\frac{R_{it}-{R}_f}{\sigma_{it}} $$

4 $$ \mathrm{Adjusted}\kern0.17em \mathrm{Sharpe}\kern0.17em \mathrm{Ratio}=\mathrm{ASR}=\mathrm{SR}i\left(1+\frac{s_k}{6}\times {\mathrm{SR}}_{\mathrm{i}}-\left(\frac{k_r-3}{24}\right)\right)\times {\mathrm{SR}}_{{\mathrm{i}}^2} $$

with *s*_k_ and *k*_r_ represent skewness and kurtosis.

5 $$ \mathrm{Sortino}\kern0.17em \mathrm{Ratio}=\frac{R_{\mathrm{it}}-{R}_{\mathrm{f}}}{\sigma {\mathrm{d}}_{\mathrm{it}}},\mathrm{with}\;\upsigma \mathrm{d}\;\mathrm{representing}\kern0.17em \mathrm{d}\mathrm{ownside}\kern0.17em \mathrm{d}\mathrm{eviation} $$

6 $$ \mathrm{Treynor}\kern0.17em \mathrm{Ratio}=\frac{R_{\mathrm{it}}-{R}_{\mathrm{f}}}{\beta_{\mathrm{it}}} $$

7 $$ \mathrm{Information}\kern0.17em \mathrm{Ratio}=\frac{R_{\mathrm{it}}-{R}_{\mathrm{m}}}{\mathrm{TE}} $$

with *R*_m_ and TE representing a return on benchmark and tracking error, respectively. Following Ayadi, Chaibi, and Kryzanowski (2016) and Clare, O’Sullivan, Sherman, and Zhu (2019), we measure the performance net of the management fees and adjust funds’ returns accordingly.

To homogenize local currencies, we translate all NAVs to the equivalent of USD at the average daily prevailing exchange rate before the computation of returns. The MSCI Emerging Markets Latin America Index is used as a market benchmark, and 10-years Brazilian Government bond is employed as risk-free. The data frequency for this research is daily. Similar to Ammann and Steiner (2009), we complement these ratios by examining Jensen’s alpha for the HCE sorted funds using Fama and French (1992) and augmented for Carhart (1997) momentum factor. Jensen’s alpha (*α*_i_) is estimated as follows:

8 $$ {R}_{\mathrm{i}}-{R}_{\mathrm{f}}={\alpha}_{\mathrm{i}}+{\beta}_{\mathrm{i}}\left({R}_{\mathrm{m}}-{R}_{\mathrm{f}}\right)+{s}_{\mathrm{i}}{\mathrm{SMB}}_{\mathrm{t}}+{h}_{\mathrm{i}}{\mathrm{HML}}_{\mathrm{t}}+{w}_{\mathrm{i}}{\mathrm{MoM}}_{\mathrm{t}}+{e}_{\mathrm{i}\mathrm{t}} $$

with SMB representing size factor, HML accounting for the book to market, *and MoM* referring to momentum. These factors are extracted from the data library (Emerging Markets) of Kenneth R. French^3^.

To establish the robustness of the impact of HCE, we use an event study methodology to differentiate the funds’ performance during Covid and pre-Covid periods. For this, we extend our data period to include 1-year funds’ returns before the global spread of Covid-19 (i.e., January 1 to December 31, 2019). Similar to Goddard, Molyneux, and Zhou (2012) and Mirza et al. (2020), this study uses the following GARCH (1,1) estimation for abnormal returns.

9 $$ {R}_{\mathrm{i}\mathrm{t}}={\alpha}_{\mathrm{i}}+{\beta}_{\mathrm{i}}\left({R}_{\mathrm{mt}}-{R}_{\mathrm{ft}}\right)+{\tau}_{\mathrm{i}}{D}_{\mathrm{i}\mathrm{t}}+{\varphi}_{\mathrm{i}}{h}_{\mathrm{i}\mathrm{t}}+{e}_{\mathrm{i}\mathrm{t}}\;\mathrm{with}\kern0.24em {e}_{\mathrm{i}}\sim {\mathrm{t}}_{\mathrm{n}}\left(0,{h}_{\mathrm{i}}\right) $$

10 $$ {h}_{\mathrm{i}\mathrm{t}}={c}_{\mathrm{i}}+{a}_{\mathrm{i}}{e}_{\mathrm{i}\mathrm{t}-1}^2+{b}_{\mathrm{i}}{h}_{\mathrm{i}\mathrm{t}-1}+{\delta}_{\mathrm{i}}{D}_{\mathrm{i}\mathrm{t}} $$

The variable *D*_it_ is the dummy that takes *t* = 1; if *t* refers to the observations during the Covid-19 contagion and *t* = 0 otherwise, *h*_it_ represents conditional variance, and *e*_it_ is the random error. The estimated parameters are *α*_i_, *β*_i_, *ϕ*_i_, *c*_i_, *a*_i_, *b*_i_, and *δ*_i_ (errors in variables). The coefficient *τ*_i_ is the estimation of the cumulative abnormal returns (CARs).

## Results and discussion

The descriptive statistics on HCE for the five sorts as of December 2019 (pre-Covid-19) are presented in Table 4. For the complete sample, the mean coefficient ranges between 2.7 (high) and 0.37 (low). Among seven countries, the equity funds in Brazil depicts maximum HCE with an average of 2.84 for the high category. This is followed by Peru and Chile that respectively have HCE of 2.77 and 2.76. In the mid-range (category 3), Argentinian equity funds have a max average HCE of 1.50, followed by those in Ecuador with a mean value of 1.47. In the low HCE category, the funds in Chile depict better efficiency than their peers with an average of 0.40.

**Table 4:** Descriptive statistics human capital efficiency base year 2019

		Low	2	3	4	High
**Overall**	Mean	0.37043	0.64687	1.16612	2.06265	2.70573
	Std Dev	0.01417	0.09001	0.15179	0.18184	0.29008
**Brazil**	Mean	0.29467	0.53417	1.10541	2.14176	2.84525
	Std Dev	0.01364	0.10034	0.11139	0.21428	0.22367
**Peru**	Mean	0.36478	0.50855	1.07772	1.53462	2.77371
	Std Dev	0.00754	0.10393	0.17548	0.17821	0.22043
**Chile**	Mean	0.40122	0.77942	1.24402	2.31118	2.76079
	Std Dev	0.01466	0.09011	0.19129	0.15988	0.23766
**Mexico**	Mean	0.39751	0.84871	1.27761	2.37314	2.74379
	Std Dev	0.01271	0.06018	0.16639	0.22235	0.34765
**Colombia**	Mean	0.37362	0.56367	1.12609	1.95300	2.60473
	Std Dev	0.01959	0.08887	0.08786	0.11247	0.38050
**Argentina**	Mean	0.15928	0.27815	1.50142	1.88691	2.16343
	Std Dev	0.00609	0.03870	0.06527	0.07819	0.12473
**Ecuador**	Mean	0.12670	0.22969	1.47531	1.92093	2.13758
	Std Dev	0.00587	0.04314	0.04790	0.09214	0.09617

**Fig. 1:**
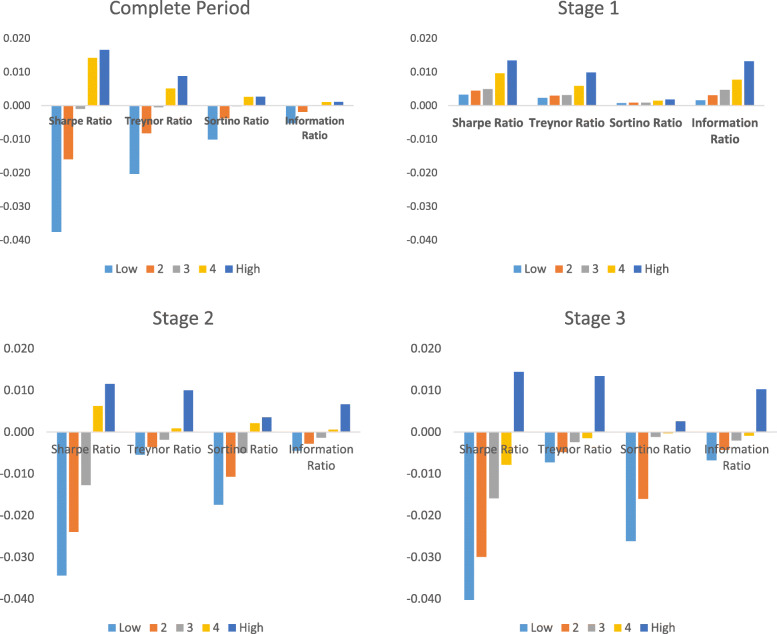
Risk-adjusted performance of funds sorted on HCE

We present our results on adjusted Sharpe, Treynor, Sortino, and Information ratios in Fig. 1. During the entire sample period, we observe that equity funds with higher HCE outperform their counterparts. It is interesting to note that funds that are low to the middle on HCE depict negative risk-adjusted performance. On the contrary, the funds in the upper sorts of HCE demonstrate positive risk-adjusted returns. The results remain consistent for the various definition of risk that includes standard deviation (adjusted Sharpe ratio), beta (Treynor), downside deviation (Sortino), and tracking error (Information ratio). The funds in the high HCE category show an adjusted Sharpe ratio of 0.016 (Treynor 0.008, Sortino 0.002, IR 0.0011). In contrast, those in the lowest HCE category demonstrate an adjusted Sharpe ratio of − 0.037 (Treynor − 0.023, Sortino − 0.01, IR − 0.005).

The stage-wise results are also presented in Fig. 1. During stage 1, the spread of Covid-19 was minimal across Latin America. Therefore, it is not surprising that our evaluation metrics demonstrate positive performance for funds included in all categories of HCE. However, it is worth noting that while all funds are in the positive zone, the performance increases as we move from low HCE to high HCE funds. The average adjusted Sharpe ratio for funds in the top HCE sort is 0.0135 (Treynor 0.0098, Sortino 0.0018, IR 0.0132). For funds in the low HCE category, the average adjusted Sharpe ratio is 0.0033 (Treynor 0.0023, Sortino, 0.0007 IR 0.0016). During stage 2, the viral infection started picking up in our sample countries. The impact is also observable in the funds’ performance. Those equity funds that are included in low to mid-HCE classification plunge into negative and this is consistent across all four performance measures. However, the funds among the two top HCE categories continue to demonstrate robust performance. The adjusted Sharpe ratio for stage 2 ranges from 0.011 for high HCE funds to − 0.043 for low HCE funds.

Nonetheless, we would like to note that albeit positive performance for funds with higher HCE, it is lower than their risk-adjusted performance in stage 1. This is plausible because as the contagion of Covid-19 was escalating, the pressures on investments were also mounting. However, despite these changing dynamics, the funds with better HCE managed to endure their performance.

Stage 3 is understandably most distressing for our sample countries. During this period, there was a massive increase in the number of cases, followed by a high mortality rate. This phase, which is still ongoing, had its toll on the financial system. We observe this impact in our results, and all equity funds except for those included in the top category of HCE have negative risk-adjusted performance. The adjusted Sharpe ratio ranges from − 0.043 in low HCE funds to 0.014 for high HCE funds. This trend is robust for Treynor and Sortino as well as Information ratios. These observations mainly reflect on the importance of human capital efficiency and how it translates into better performance for funds. While all other funds were negatively impacted, the high HCE funds endured resilience and continued to perform through these turbulent times.

**Fig. 2:**
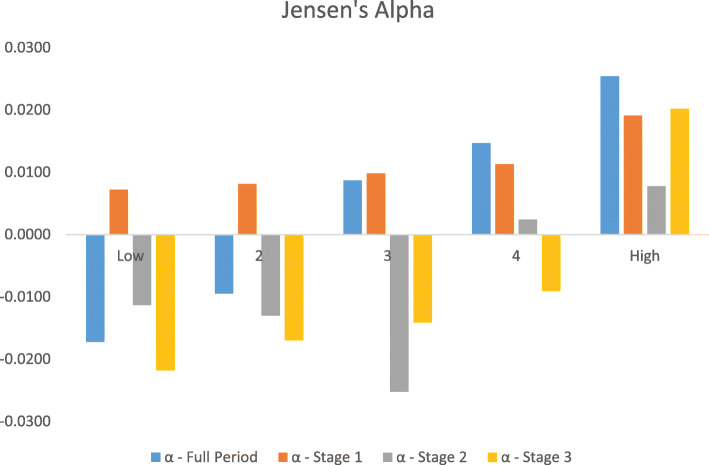
Jensen’s alpha for HCE sorted equity funds

The results for Jensen’s alpha are included in Fig. 2. For the entire period, we observe negative alphas in the two lowest HCE category of funds. However, the funds contained in medium to high HCE posit positive excess returns. The maximum alpha is reported for the top HCE funds signifying that human capital efficiency translates into performance. The observed alphas for the entire period across the five HCE sorts are significant at 1%. For stage-specific alphas, the story is similar to our findings from adjusted Sharpe, Treynor, Sortino, and Information ratios. During stage 1, all funds depict positive alphas with maximum excess returns for funds in the high HCE category. In stage 2, funds in three (low to medium HCE) out of five categories have negative alphas while top HCE funds remained persistent with maximum alpha. Finally, in stage 3, the funds in the high HCE category demonstrated positive alpha while all others have negative excess returns. The results are statistically significant at 1% and 5%.

The results for GARCH-based event study methodology are presented in Table 5. We observe profound differences in CARs for the pre-Covid and Covid-19 periods. During the pre-Covid period, only the topmost HCE category funds show positive abnormal returns, while for the rest, we document negative CARs. During the outbreak period, we see a significant increase in positive CARs for the top two HCE category funds. On the contrary, the funds included in the lower HCE categories experienced a further deterioration in the performance with higher negative CARs. These findings suggest that while HCE is relevant in general, during the Covid-19 period, its importance for the performance of equity funds in Latin America has increased manifold.

**Table 5:** Abnormal returns of HCE sorted funds prior to Covid-19 and during outbreak

Fund type	Average cumulative abnormal returns GARCH (1, 1)	
	Pre-Covid	Covid outbreak
**Low**	− 0.0161%^b^	− 0.0202%^a^
**2**	− 0.0122%^b^	− 0.0170%^b^
**3**	− 0.0059%^b^	− 0.0063%^b^
**4**	− 0.0205%^b^	0.0391%^a^
**High**	0.0251%^a^	0.0701%^a^

^a^Significance at 1%

^b^Significance at 5%

The overall results support our a priori notion of the impact of human capital efficiency with the varying performance of funds according to their HCE. The funds that performed persistently are the ones with superior HCE, and degradation in performance is observed as we move from high to low HCE funds. It is worth noting that this is a period when global financial markets are in rout amid the outbreak of Covid-19. Therefore, it is remarkable that some funds continue to remain resilient in these turbulent times, and we attribute this resilience to their human capital efficiency.

## Conclusion

The importance of human capital efficiency is central in financial services like funds management, where performance is driven by investment strategies devised by the portfolio managers. While many studies evaluate skills vs. luck in mutual fund returns, there are not many that have assessed the role of human capital efficiency. In this study, we attempt to fill this gap by evaluating the risk-adjusted performance of mutual funds in seven Latin American states by ranking them as per their human capital efficiency. The sample period that overlaps with the outbreak of Covid-19 provides a unique perspective on the performance of HCE sorted funds as these 6 months are akin to an economic crisis. Our findings suggest that during these stressed times, funds with higher human capital efficiency tend to outperform their counterparts. This phenomenon remained consistent while the Covid-19 continued to escalate, and funds with lower HCE experienced significant performance deterioration. While these findings present an essential aspect of the performance of Latin American funds, the situation surrounding the Covid-19 is very dynamic. Therefore, it will be necessary to continuously evaluate the HCE sorted funds to generalize these results over the medium to long term.

## Data Availability

The datasets generated and/or analyzed during the current study were hand collected by the authors and available from the corresponding author on reasonable request.

## Abbreviations

HCE: Human capital efficiency
NAV: Net asset value
WHO: World Health Organization
SMB: Small minus big
HML: High minus Low
Covid-19: New coronavirus

## Footnotes

1 Semi Annual Global Economic Perspectives by World Bank (June 8th 2020)

2 On January 1, a new infection was formally reported to WHO that was later named as Covid-19

3 The data library is accessible at http://mba.tuck.dartmouth.edu/pages/faculty/ken.french/data_library.html
